# Enhancing a Client-Centred Practice with the Canadian Occupational Performance Measure

**DOI:** 10.1155/2018/5956301

**Published:** 2018-06-27

**Authors:** A. Enemark Larsen, B. Rasmussen, J. R. Christensen

**Affiliations:** ^1^Department of Occupational Therapy, Institute of Medwifery and Therapy, University College Copenhagen, Sigurdsgade 26, DK-2200 N Copenhagen, Denmark; ^2^Department of Public Health, University of Southern Denmark, JB Winsløwsvej 9A, 5000 Odense, Denmark; ^3^Department of Sports Science and Clinical Biomechanics, University of Southern Denmark, Campusvej 55, 5230 Odense, Denmark

## Abstract

**Background:**

The active participation of clients is an important aspect of rehabilitation quality as conceptualized in client-centred practice (CCP). A recommended outcome measure for enhancing CCP is the Canadian Occupational Performance Measure (COPM). However, the relationship between COPM use and CCP enhancement has not been documented.

**Aim:**

The aim of this study was to examine whether the use of the COPM enhanced CCP.

**Methods:**

We performed a scoping review in five steps: (1) *identifying* a search strategy with inclusion and exclusion criteria; (2) *screening* relevant databases for published and unpublished studies by using selected keywords and by manually scrutinizing reference lists; (3) agreeing on *eligible* papers between authors in terms of inclusion and exclusion criteria; (4) charting *included* data; and (5) *analysing* data using qualitative content analysis.

**Results:**

Twelve studies were included in the review. The results indicated enhanced CCP in two themes when using the COPM. These themes appeared to influence each other; therefore, the first theme, *Conditions for enhancing CCP when using the COPM*, represented the circumstances needed for the second theme, *Enhancing CCP when using the COPM*, to be fulfilled.

**Conclusion:**

The use of the COPM seems to enhance CCP if certain conditions are present.

## 1. Introduction

The active participation of clients is an important aspect of the quality of rehabilitation processes and the aim in healthcare institutions around the world [1, 2]. Similar in Denmark, since 2013, the hospital staff have endeavoured the active involvement of clients through a systematic collection of information from clients about their experiences during rehabilitation [3]. The information has been collected within the Danish Model of Quality in the Healthcare System (DDKM) and used to improve the quality of hospital services [3]. Thus, active participation of clients is included as a key component in the 2020 visions of healthcare systems in many countries [4]. The 2020 visions aim to create a healthcare system that is suited to each individual and more responsive to each client's preferences, needs, and values [4]. However, fulfilling this aim is difficult [5, 6]. A recent study showed that although the aim is the active participation of clients in the rehabilitation process, the process often does not truly reference the client's wishes and goals and is not collaborative [7]. Another study showed that clients experienced inadequate rehabilitation that did not emphasize their individual and emotional needs [8]. Furthermore, clients who require help to manage uncertainties have experienced unaddressed problems [8, 9]. All this evidence illustrates the need to optimize efforts to enhance client participation in the rehabilitation process [10].

One conceptual framework that includes the active participation of clients is “client-centred practice” (CCP), described by Carl Rogers as practice that is nondirective and focuses on the concerns expressed by the client [6, 11]. In occupational therapy (OT), CCP has always been an integrated value and a fundamental element [12]. Research on CCP has increased over the last few decades [6, 13–15]. A systematic review revealed five thematic conceptual elements that should be addressed when working with CCP, namely, power, listening and communicating, partnership, choice, and hope [6].

A recommended method believed to enhance CCP in the rehabilitation process is the use of the Canadian Occupational Performance Measure (COPM) [16]. The COPM is designed to identify changes in the client's personal perceptions of occupational performance over time, and studies show that the identification of self-perceived occupational performance problems (OPPs) seems to enhance client motivation and the relevance of the individualized goals in rehabilitation [16–20]. The COPM consists of the following five steps and three scores: (1) the clients identify their OPPs within the areas of self-care, productivity, and leisure and score their *importance* on a scale ranging from 1, not important, to 10, extremely important. (2) The clients prioritize up to five OPPs on which to focus during the intervention. (3) The clients rate their *performance* and (4) their *satisfaction with performance* of the identified OPPs. Both scales range from 1 to 10, with higher values indicating better performance and greater satisfaction. (5) Performance and satisfaction with performance are reassessed after an appropriate interval of time, normally between three to six months, and changes in the clients' perceptions of their occupational performance can be calculated [16].

Generally, studies on concurrent and content validity that included a variety of clients and OT practitioners show that the COPM is a valid measure of occupational performance [20]. Studies have also documented the COPM's utility in providing an individualized perspective of the client's values, judgements, and preferences regarding occupational performance and thus its ability to facilitate clinical decision-making and monitor functional progress [19, 20].

Given that the aim of the COPM interview is not only to identify, prioritize, and score the OPPs but also to enhance CCP, it seems important to evaluate whether the latter aim is actually fulfilled. Studies of the COPM have also reported the ability of the COPM interview to enhance CCP [17–20]. Additionally, in the systematic review by Parker and Sykes, the COPM interview enabled clients to name and frame their problems, allowed them to be experts on their own circumstances, and empowered them to be more critical of their own performance, that is, the use of the COPM seemed to support CCP [21]. However, a review from 2011 that examined the COPM and other measurements identified a paucity of literature concerning the client's experiences with the COPM, leaving the question of whether the use of the COPM can enhance CCP [22]. Thus, the aim of the current study was to examine the literature and determine whether the use of the COPM can enhance CCP.

## 2. Materials and Method

### 2.1. Design

The current scoping review followed the recommendations from the commentary of Colquhoun et al. [23], which advised the use of the following methodological steps outlined in the Arksey and O'Malley framework [24] and further enhanced by Levac et al. [25]: (1) identification: identify the research questions. (2) Screening: find the relevant studies. (3) Eligibility: select the studies relevant to the question. (4) Inclusion: chart the data. (5) Analysis: collate, summarize, and report the results. Because our aim was to examine the *literature* on the COPM, the optional 6th step of the scoping review procedure, consultation with stakeholders as a required knowledge translation component, was not applied in this review [23–25].

### 2.2. Procedure

#### 2.2.1. Identification

We developed a search strategy with inclusion and exclusion criteria and used the PICO (population, intervention, comparison, outcome) model to identify keywords relevant to our research question [26] as we wanted to examine whether the COPM (the ‘I') would enhance CCP (the ‘O'). Thus, in our search strategy, we included all kinds of populations from all kinds of settings (the ‘P's), but we did not include any comparisons (the ‘C').

#### 2.2.2. Screening

The literature search was performed in two steps between September and December 2016 (last search: 18.12.2016). First, an initial literature search in PubMed and CINAHL was performed to identify relevant keywords, synonyms, word modifications, and thesaurus terms to use to adjust the O criteria—the clarification of the term “client-centred” [26]. Several searches using different word combinations were performed until we found a search strategy that seemed to identify studies on the COPM and CCP. Keywords were narrowed to the framework of “client-centred” as this is the term used in the COPM and the OT profession, for which the COPM was developed. Other concepts such as “patient-centred” or “patient-focused care” were not included, because although similar, they define an organizational process rather than a conceptual approach to ensure the participation and engagement of clients. Thus, we used the following keywords: ((COPM) OR “Canadian Occupational Performance Measure”) AND ((client-center^∗^) OR client-centre^∗^)).

Second, database-specific searches using PubMed, CINAHL, OTseeker, PEDro, Web of Science, and SCOPUS were conducted using the thesaurus terminology for each database. Truncations were used when relevant. The abovementioned keywords were also used to search for unpublished items on interventions of interest in SwePub and NORA. Google Scholar was also examined using the terms “COPM” and “CCP”.

#### 2.2.3. Eligibility

The selection of papers eligible for inclusion was guided by inclusion and exclusion criteria based on the research question. The papers were selected by agreement among the authors. The *inclusion criteria* were the following: (I) studies of all kinds of interventions in all settings, addressing all adult clients ≥ 18 years to whom the COPM had been administered. (II) Studies that revealed the OTs' or their clients' experiences with the use of the COPM to enhance CCP. (III) Published and unpublished qualitative and quantitative studies. (IV) Studies published from January 2005 to August 2016 as the research on the COPM and CCP has increased over the past decade. The *exclusion criteria* included the following: (I) studies in which the COPM only was used as an outcome measure, without data on the participants perception of the use of the COPM. (II) Studies focussing on children. (III) Studies written in languages other than English, Danish, Swedish, or Norwegian. (IV) Editorials, commentaries, interviews, lectures, periodicals, and abstracts.

The two first authors conducted the study selection. Agreement was reached by discussion between the authors, and the third author was consulted if unresolved disagreements occurred. All studies were entered into an Excel sheet to identify and exclude duplicates. The titles and abstracts of the remaining studies were assessed for the inclusion and exclusion criteria, thus facilitating the exclusion of studies that did not fit the research question. The remaining studies were read in full, and studies that were unfit to answer the research question were excluded. Finally, the reference lists of the studies that were read were examined to identify additional studies eligible for inclusion in the scoping review.

#### 2.2.4. Inclusion

As recommend by Levac et al. [25], the authors collectively conducted first assessment of the included studies to determine which descriptive variables to extract to answer the research question. Because the included studies were mostly qualitative, we followed the recommendations of Levac et al. and used a qualitative content analysis approach [25, 27].

#### 2.2.5. Analysis

The qualitative content analysis was based on the recommendations of Graneheim and Lundman, focussing on latent context analysis centred on an in-depth interpretation of the underlying meaning of the text [27]. The two first authors performed the analysis. The included studies were read independently to obtain a general sense of the entire pool of included studies. Then, each author performed a systematic identification of textual information, located the important structures and information from each study, and entered these textual units into a table to identify how these “constellation of words” related to the central meaning. The two tables were compared, and the differences were discussed to subsequently form a common table of significant “meaning units” that had been identified and agreed upon. To ensure that all relevant content was identified, the authors reread all studies while searching for text in accordance with the identified “meaning units” and entered new findings into the table. The content of the meaning units was condensed and abstracted to identify adequate labels, or codes, to represent each condensed meaning unit. The authors compared the codes to identify differences and similarities and sorted the codes into themes and subthemes related to the research question. Finally, all studies were reread to ensure that all the information had been extracted into the themes and subthemes, and dialogues with the third author ensured that nothing in the studies contradicted the themes [27].

## 3. Results

**Figure 1:**
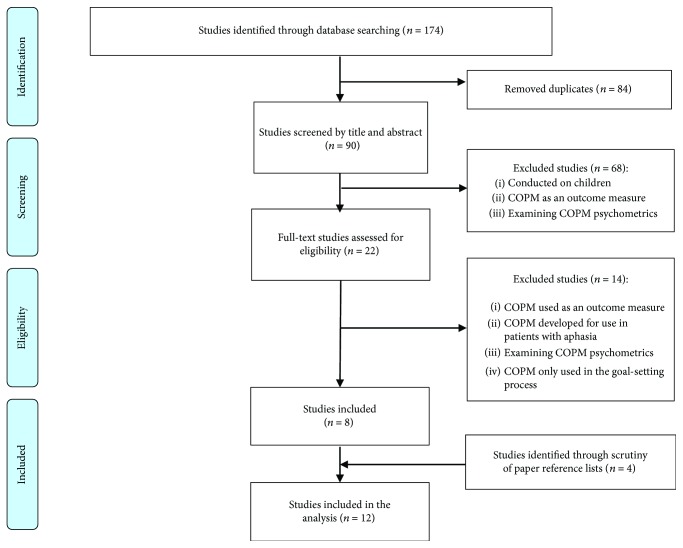
Study flow.

As presented in Figure 1, we identified 174 studies, and 90 studies remained after duplicates were removed. After the titles and abstracts were screened, 22 studies remained for full-text readings. The authors independently read the 22 studies and discussed their inclusion and exclusion, which left eight studies. After a careful examination of the reference lists of the eight studies, four studies were added.

The twelve studies included one systematic review [28], one opinion paper [29], one quantitative study [30], four qualitative studies [1, 31–33], and five mixed-method studies [19, 34–36], including one unpublished PhD dissertation [11]. The contents of each study, including their aims, methods, samples, and settings, are summarized in Table 1.

The analysis of the literature on the ability of the COPM to enhance CCP revealed two main themes, with five subthemes in the first theme and four subthemes in the second. The two main themes seemed to influence and affect each other; thus, the first theme represented the circumstances required for the second theme to be fulfilled. The first theme considered the prerequisite conditions for CCP when conducting a COPM interview. Some of these conditions related to the OTs' skills and attitudes, specifically in relation to *communication* and *willingness to listen to the clients' points of view*. Others related to how *the environment* seemed to affect the ability of the COPM interview to enhance CCP, particularly in terms of the institutional *commitment to CCP* and the willingness to *share the power with the client*.

The second theme concerned the aspects of CCP that a COPM interview seemed to enhance. These aspects included how the interview could lead to an increase in the OT's awareness of the client's preferences, needs, and values, such as that the OT *got to know the client* and was able to *enhance the client's self-awareness*. Additionally, the use of the COPM interview seemed to enable the processes of *forming a partnership* and *develop collaborative goals*. When these aspects of CCP occurred, they seemed to positively strengthen and increase the OT's behaviours and the environmental conditions under which the COPM was administered. The themes are narratively presented, and an example of the analysis and translation from meaning units to themes is shown in Table 2.

### 3.1. Conditions for Enhancing CCP When Using the COPM

Our analysis of the included studies indicated that certain conditions should be met if CCP is to be enhanced by the use of the COPM. The OTs' skills and their willingness to *communicate with* and *listen to* the clients' points of view are necessary for the COPM to enhance CCP [11, 19, 30, 31, 33, 36]. A statement by one of the OTs in one of the studies supported the importance of these factors: “It takes very expert communication skills to use [the COPM] in the way it is designed and for it to actually have a therapeutic benefit” [33]. Tuntland et al. reported that personnel without bachelors-level qualification felt a need for additional training in the COPM interview and CCP, indicating that they regarded their expertise as insufficient [30]. Stevens et al. focused on the OTs' communication skills: “It's about the skillful interview in the initial stages and communication to establish what the client is having difficulties with and negotiate with them the areas they want to work on... being honest with them … [and] provide and renegotiate … goals…” [28].

Other studies indicated that for the COPM interview to facilitate CCP, a *commitment to CCP* that included an acceptance of the client's opinion, that is, *sharing the power*, was important [1, 11, 19, 28, 29, 33, 36]. Further, the OT's reluctance to use the COPM and his or her commitment to CCP could be interpreted as depending on *the environment* in which the rehabilitation occurred. For instance, if the OT focused on restoring physical function while working in a hospital, contradictory paradigms such as the traditional biomedical model would be inconsistent with the use of the COPM, which is based on a holistic approach [1, 19]. It seemed that therapy was influenced by the environmental conditions of the respective institutions and that this could lead to differences in expectations between clients and OTs [1, 19, 28, 36]. The OTs felt uncomfortable having to listen to and go along with their clients and expressed ambivalence when dealing with the clients' wishes if these wishes differed from the OTs' own [1, 19, 28]. This process was difficult if the OTs found the clients' goals unrealistic as the OTs felt they had the expertise to know what was best for the client [1, 28]. Stevens et al. found that “Some [OTs] indicated that involving patients even undermined the therapy and reduced the patient's motivation, reporting that they [the patients] set unrealistic goals or had unrealistic expectations” [28]. Thus, in some settings, an OT's reluctance to use the COPM to determine the client's point of view and to power were identified as impediments [1, 28], and discrepancies between client aspirations and OT expectations were observed [1, 19, 32, 36]. In a survey on CCP, Parker found that although only 6% of participating OTs agreed with the statement that “the therapist should set the treatment goals,” 27% of the OTs agreed that “the therapist has different goals than those of the client” [11]. In the studies by Richard and Knis-Matthews [1] and Engelbrecht et al. [36], differences in the goals of the therapy were observed. The clients identified goals outside the context of the residence programme, such as returning to work or school, whereas the OTs focused on achieving goals within the residence programme [1]. In these studies, the OTs' ideas of feasibility seemed to limit collaborative goal setting [1, 36]. These limitations were related to the clients' constitutions, for example, if the clients had just become ill or did not have the cognitive ability to conduct a COPM interview, or the environmental conditions, for example, what the OTs were expected to address or the available facilities [19, 28, 30, 35, 36]. However, if the environmental values aligned with CCP, the OTs seemed to translate the client requirements into goals based on the COPM interview [19].

### 3.2. Enhancing CCP When Using the COPM

In all the studies, there was agreement regarding the benefits of using the COPM, most frequently expressed as the ability that the COPM provided for the OTs *to get to know the client*. This awareness was two sided; the OTs familiarized themselves with the clients and their aspirations, and *the clients gained better insight* and awareness of what they wanted and what to expect from the intervention. This often *facilitated partnerships* in the CCP through *collaborative goals*.

It seemed that the use of the COPM provided a positive starting point for CCP because it improved the OT's knowledge of the client's everyday life and aspirations and encouraged the collaborative formation of goals based on the identified OPPs [19, 28, 30–33]. Apparently, using the COPM could emphasize the importance of clinical judgement for guidance [31, 33]. In the study by Donovan et al., the use of the COPM increased equality in the therapeutic relationship by enhancing the understanding of individual clients' perspectives and shifting perceptions, attitudes, and behaviours towards occupations that are meaningful in the clients' lives [32]. In the study by Tuntland et al., the OTs considered the COPM to be very useful in planning and evaluating the intervention [30]. The majority (82%) of the clients stated that they felt the COPM interview and scoring were useful [30].

In addition to the OT's positive experiences with the COPM, the COPM also seemed to improve interdisciplinary teamwork around the client, providing broader information regarding the client, increased information on occupational performance, and a greater emphasis on team goal setting [11, 19, 28, 30].

Colquhoun et al. found evidence that OT knowledge of what matters to the client shows statistically significant improvements when using the COPM [34]. In hand therapy, the use of the COPM was thought to help the therapists focus on issues and goals that would assist the client in fulfilling important life roles [29].

Tuntland et al. found that the COPM was useful for determining goals, and clients felt that the COPM contributed information about “what is important to me” [30]. The COPM interview encouraged the clients to increase their attentiveness to their problems and enhanced their motivation, cooperation, and responsibility for their own therapy [28, 31], thus facilitating a process of self-awareness [19, 32]. The COPM interview seemed to improve the clients' understanding of their own issues, making difficult experiences in everyday life tangible, and thereby facilitated the client-centred partnership [31, 33]. As the COPM interview increased awareness of daily life and acknowledged the clients [30], it helped improve the degree to which the rehabilitation was client centred [33]. The importance of being acknowledged was also supported by Parker, in whose study clients expressed the overwhelming importance of how they were treated [11]. The clients expressed a fundamental need to be valued as individuals, indicating that the attitudes of the OTs in these relationships are more important than their actions [11].

## 4. Discussion

The assumption that the use of the COPM facilitates CCP led to the objective of this study to examine the literature to determine whether CCP is enhanced when the COPM is used. The scoping review found that the use of the COPM seemed to enhance CCP and that certain conditions were necessary for the COPM interview to have this desired effect.

### 4.1. The Use of the COPM Seemed to Enhance an Awareness That Goes beyond Assessments

The process of identifying OPPs seemed to change the clients' awareness of their self-perceived needs, which further appeared to affect the client's motivation, cooperation, and responsibility through the subsequent rehabilitation. The COPM interview seemed to influence the clients' attentiveness to and understanding of their problems, which seemed to facilitate a process of self-awareness, making difficult experiences in everyday life more tangible. Thus, starting a rehabilitation process with a COPM interview might help the client begin to come to terms with life. In this way, the COPM interview appeared to initiate a process similar to what Antonovsky called a “sense of coherence” (SOC) [37]. Studies have shown that a SOC contributes to a client's quality of life, health, and satisfaction with daily occupations [37, 38]. Provided that the COPM interview has this positive influence on the client's self-awareness, the COPM interview might facilitate CCP beyond the assessment by influencing the rehabilitation outcome itself.

### 4.2. The Use of the COPM Helped with Forming a Partnership and Making Collaborative Goals

The COPM interview led to an increased awareness of the client's preferences, needs, and values and helped the OT and the client form a partnership with meaningful and collaborative goals [11, 19, 28, 29, 31–33]. An important aspect of CCP is the establishment of a client-centred partnership in which the client plays an active role in defining goals [6]. Forming a partnership with clients and collaborating in goal setting have always been valued processes in OT [5, 6, 11, 12, 39, 40] that are described as ethical values in both national and international OT associations [41, 42]. Because partnerships and collaborative goalsetting have a positive effect on the outcomes of rehabilitation [15, 43], these aspects are important parts of healthcare politics and have formed the basis for many government policies in recent years [4, 44].

This scoping review revealed that using the COPM to examine client perceptions might help to minimize the OTs' misinterpretation of client needs, goals, and wishes [1, 32]. The included studies showed a discrepancy between goals of the OTs and those of their clients. Similar discrepancies between therapists and clients have been seen in other studies, for example, in stroke rehabilitation [15, 43]. Here, the OTs prioritized goals characterized by short timeframes, conservative estimates of outcomes, and physical function, that is, focused on impairments, independent self-care, and locomotion [15, 43], whereas the clients focused on resuming their predisease status and participating in social activities [15, 43].

### 4.3. Environmental and Therapeutic Factors Seemed to Affect If a COPM Interview Enhanced CCP

This review showed that the OTs' commitments to CCP, their willingness and ability to share power with the client, and the environment in which the rehabilitation occurred were important factors determining whether the COPM interview would positively affect CCP. Environmental factors affected the possibility of conducting a COPM interview as the OTs were reluctant to perform a COPM interview if the focus was on physical functions, that is, in settings where a medical paradigm was dominant [15, 19–21]. Some of the reluctance seemed to be an attempt to avoid the dilemma of different goals. This was also seen in the study of Mortenson and Dyck in which the OTs avoided the use of the COPM because they feared it would reveal differences between what the clients wanted to achieve and what the OTs were expected to deliver [45]. Other reasons for not using the COPM were that the clients had suddenly became ill or been hospitalized or that they did not have the cognitive ability to undergo a COPM interview [21]. When clients experience an acute disease, a preoccupation with the body's condition, symptoms, and prognosis may follow [46, 47], in addition to feelings of loss, decreased self-esteem, frustration, and anger [48]. Accordingly, in the early stages of a rehabilitation process, clients may ignore or be unaware of OPPs, which explains why it might be difficult to use the COPM under such circumstances [19, 21, 47]. However, the OTs' disinclination to use the COPM in the early stages of their clients' illnesses may reflect CCP in which the OTs acknowledged their clients' fragility and use of counselling for client-centred care [49]. Thus, although the COPM interview seems to enhance CCP, it can be argued that sometimes CCP is better provided without the COPM.

However, some OTs' reluctance to implement CCP could be due to the notion that the OTs considered themselves the experts and thus believed that they knew what was best for the clients. This illustrated a contradiction of paradigms, for example, the traditional biomedical model, with the therapist as the expert, versus the occupational model of the COPM, the Canadian Model of Occupational Performance (the CMOP), in which the client is considered the expert [12, 16]. It seems that the more therapists align with the medical paradigm for example, in hospitals, the lower their use of the COPM interview [15, 19]. This was also the case in a study of implementing the COPM in Taiwan. In that study, practice was driven by the biomedical model; thus, the concepts of COPM and CCP seemed unfamiliar and required adjustments from both therapists and their clients [49].

### 4.4. The COPM Interview Requires Advanced Communication Skills

Effective communication skills appeared to be vital to the successful use of the COPM and the enhancement of CCP. Corresponding findings have been reported in other studies on CCP, which describe a focus on interviewing skills, for example, the ability to listen actively, communicate in an appropriate language, and share information that aligns with the clients' needs [4–7, 20, 21, 38, 39]. Studies on how to support the successful implementation of the COPM have shown that introductory courses, ongoing support, and follow-up supervision during the implementation process are beneficial [19, 33–35]. Regular reviews might also be beneficial since Colquhoun et al. found that despite support, sustained use of the COPM was not observed [35].

#### 4.4.1. Methodological Considerations

Despite rigorous measures, such as using two reviewers for all sources, this study has limitations that are inherent to scoping reviews in general, such as the synthesis of both published and unpublished studies and qualitative and quantitative research to balance broadness and depth. Since a scoping review is not meant to be exhaustive but rather to provide a good sense of the literature in a particular area, it is likely that some relevant publications were not included in this study. Moreover, given the global nature of the COPM, the exclusion of studies published in languages other than English or Scandinavian makes it unlikely that all relevant studies are included. The number of studies may also have been limited because we narrowed our keywords to the framework of “client-centred,” the term used by the COPM and the OT profession. However, by screening the references of the included articles, we attempted to limit the number of missed articles.

## 5. Conclusion

This scoping review examined whether the use of the COPM could enhance CCP. Twelve studies were identified that used different designs and a range of clients in various settings. The review found that the use of the COPM seems to enhance CCP, specifically by improving awareness of client perspectives, wishes and hopes for the future, thereby reinforcing a partnership with collaborative goalsetting. However, to use the COPM interview to enhance CCP, professional communication skills, power sharing, and an institutional commitment are required.

## Figures and Tables

**Table 1:** An overview of the included papers (*n* = 12).

Reference name/number	Goal/aim	Study design	Method	Materials (sample and context)
Bjørkedal et al. [31]	To qualitatively evaluate the feasibility of client-centred OT intervention focused on enabling meaningful occupations and supporting the recovery process for individuals with schizophrenia in the early phases of recovery.	Qualitative study.	A qualitative study comprising an eight-week client-centred OT intervention and semistructured interviews with five of the six clients out of 10 who completed the intervention. The COPM was used to initiate and guide the intervention, and the Canadian Model of Client-Centred Enablement was used for the client-therapist relationship.	Ten participants with schizophrenic disorders, five of whom (three women and two men) participated in postintervention semistructured interviews. They were recruited from the community and lived in their own apartments.

Colquhoun et al. [34]	To survey OTs currently using the COPM to gather their perceptions about the use of the COPM on a routine basis. A secondary objective was to propose a meaningful template for summarizing routine COPM data.	Mixed method study.	The OTs were given a short-answer written questionnaire that focused on the key areas, derived from the literature, in the routine outcome measures used. These questions related to the feasibility of the COPM and the value of the data. In addition, the resulting five months of COPM data were summarized into a proposed template based on what the OTs found meaningful.	Three female OTs, with six, eight, and 31 years of practice. They had been working in the geriatric unit for one year, five years, and one year, respectively.

Colquhoun et al. [35]	To determine whether COPM administration was associated with changes in eight dimensions of OT practice.	Mixed-method study.	A before-and-after study with repeated tests. The eight practice dimensions were assessed after three months of usual care (no COPM use) and after three months of intervention (COPM use) using chart-stimulated recall (CSR) interviews and chart audit.	24 OTs working in eight inpatient geriatric rehabilitation facilities across two large urban centres. Potential sites were identified by geographic area and the presence of inpatient geriatric rehabilitation units.

Donovan et al. [32]	To describe the occupational concerns and goals of mothers who care for children with disabilities.	Qualitative study.	A qualitative design analysing retrospective data collected using the COPM.	38 mothers of children with disabilities.

Enemark Larsen and Carlsson [19]	To evaluate the utility of the COPM in an interdisciplinary geriatric rehabilitation context (RCC) in Copenhagen in terms of (a) its utility to both OTs and physiotherapists, (b) its utility to document change, and (c) the therapists' experiences with the administration and usability of the COPM.	Mixed-method study.	Data were collected from a pre- and postassessment with the COPM and a questionnaire answered by all participating therapists.	18 therapists (11 physiotherapists and seven OTs) participated in the project group. 185 elderly clients referred to the RCC by home care personnel or general practitioners participated in the study through consecutive sampling over a period of eight months.

Engelbrecht et al. [36]	To determine whether healthcare workers could successfully identify the occupational performance priorities of adult people with disabilities (PWD) living in a Kwaguqa community in the Mpumalanga province of South Africa.	Mixed-method study.	A nonexperimental descriptive design using descriptive survey questionnaires, for example, the COPM, which captured quantitative and qualitative data.	25 adults (17 men and 8 women) from the Thembelihle Self Help Centre and the Kwaguqa Association for the Disabled Workshop with mobility or dexterity problems, aged 18 to above 60 years of age, plus seven female healthcare workers responsible for the service in the Kwaguqua area.
Gustafsson et al. [33]	To investigate the goal-setting process and clinical utility of the COPM from the perspective of OTs within a spinal cord injury unit (SIU).	Qualitative study.	A focus group interview was conducted with seven OTs working in an SIU to explore their experiences and use of goalsetting and the COPM. Inductive thematic analysis identified key themes from their comments.	Seven female OTs, with a mean age of 29.7 (SD 8.38) years. Their clinical experience working in the hospital SIU ranged from less than 6 months to more than 20 years.

Hannah [29]	To summarize the psychological impact of severe hand injuries, to discuss coping strategies for the social impacts of severe hand injuries, and to outline assessments and strategies that can be used by hand therapists to treat the whole person, develop patient-centred goals, and improve therapy outcomes.	An opinion study based on a literature review.	Opinion based on a literature review.	No material included as due to the study format.

Parker [11]	To determine clients' and OTs' perceptions of the CCP in OT in the UK	PhD dissertation. Different studies—a review, a survey, and a qualitative design.	A mixed-method study examining the views of clients and therapists was undertaken using a systematic review to examine global evidence of client-centred outcome measures, a survey of the experiences of a sample of therapists and individual client and OT interviews.	Study (1): 25 OTs opted to attend the focus group. The gender profile of the group was 3 males and 22 females. Study (2): 230 questionnaires were sent to OTs, and 25% were returned. Study (3): four OTs with 4–30 years of education and four clients aged 35–81 years.

Richard and Knis-Matthews [1]	To compare the intervention goals identified by clients and an OT using the COPM.	Qualitative study.	In-depth interviews were conducted with an OT and her clients in conjunction with the use of the COPM.	Seven clients living in a long-term residential programme and diagnosed with schizophrenia and one OT who had worked at the programme for three years at the time of the study.

Stevens et al. [28]	To identify the currently available patient-specific measurement instruments used in the process of goalsetting and to assess their feasibility.	A qualitative systematic review.	After a systematic search in PubMed, EMBASE, CINAHL, PsychINFO, and REHABDATA, patient-specific instruments were included, structured within a goal-setting practice framework and subjected to a qualitative thematic analysis of feasibility.	25 patient-specific instruments were identified, and 11, one of which was the COPM, were included.

Tuntland et al. [30]	To investigate the validity, responsiveness, interpretability, and feasibility of the COPM when used by various health professions in home-dwelling older adults receiving assistance.	A quantitative study on psycometrics.	A quantitative study following the COSMIN guidelines and recommendations for evaluating methodological quality.	The sample included 225 participants, mean age 80.8 years, in need of rehabilitation for various health conditions. Data collection was conducted at baseline and at a 10-week follow-up. The assessments were conducted by 12 nurses, 33 OTs, 27 physiotherapists, five auxiliary nurses, and one social educator.

**Table 2:** Meaning units, condensed meaning units, subthemes, and themes from the content analysis.

Examples of meaning units	Condensed meaning unit/interpretation of the underlying meaning	Subthemes	Theme
The participants found that the COPM made the challenges and barriers that they experienced in their everyday lives tangible, helping them set suitable personal goals and initiate the intervention ([31], p. 102).	The COPM helped the clients to comprehend their everyday lives and set suitable goals for intervention.	*Getting to know the client* and *Enhancing clients' self-awareness*	*Enhancing CCP with the COPM.*
The COPM proved to be a suitable measure for determining occupational performance priorities from the perspectives of both the PWD and the healthcare workers (HCWs), although this initially resulted in diverse lists of priorities ([36], p. 13).	The COPM helped the clients and the OTs identify OPPs. The identified OPPs differed between clients and OTs.		
The OTs felt that the COPM facilitated discussions with clients about functional independence and the formulation of OT-related goals. They felt that until they actually performed the COPM with a person, they did not really have a sense of the person's needs. The COPM was also thought to aid in increasing client motivation and the client's sense of control over their rehabilitation; it made the rehabilitation process meaningful for the client and provided OTs with client-centred directions and priorities for therapy. An OT stated, “[The COPM] also assists in your assessment of knowing the person's insight and adjustment to their disability” ([33], p. 340).	The COPM helped the OTs get to know their clients. The COPM also helped the clients and facilitated insights for both the OTs and the clients.		
The participants felt that the COPM interview led to greater awareness of their daily lives and to a feeling of being seen and listened to. They also reported that the interview enhanced their motivation to focus on improving occupational performance and that the information brought forward during the interview and scoring process was useful as a basis for developing rehabilitation goals ([30], p. 419).	The COPM led the clients to a greater awareness of their daily lives and enhanced motivation and development of goals.		
